# A Case of Near-Fatal Bradycardia Caused by Accidental Cannabis Intoxication

**DOI:** 10.7759/cureus.37430

**Published:** 2023-04-11

**Authors:** Shelby Ploucher, Sarina Koilpillai, Murali Iyyani, Steve Carlan

**Affiliations:** 1 Internal Medicine, Orlando Regional Medical Center, Orlando, USA; 2 Obstetrics, Orlando Regional Medical Center, Orlando, USA

**Keywords:** drug-induced bradycardia, in-hospital cardiac arrest, geriatric medicine, cannabis legalization, synthetic cannabis

## Abstract

The recreational and medicinal uses of cannabis are increasing worldwide. Given the recent legalization of marijuana in some regions of the United States, the use of edible formulations has become increasingly popular, especially among the elderly. These new formulations can be up to 10 times more potent than previously available preparations and have been associated with a variety of cardiovascular adverse effects. Here, we present a case of an elderly male who presented with dizziness and altered mental status. He was found to be severely bradycardic and emergently required atropine. Further investigation revealed that he accidentally ingested large amounts of oral cannabis. An extensive cardiac workup revealed no other etiology for his arrhythmia. Cannabidiol (CBD) and tetrahydrocannabinol (THC) are the most commonly studied cannabis compounds. With the increased access to and popularity of edible cannabis formulations, this case demonstrates the need for further research regarding the safety of oral cannabis.

## Introduction

Marijuana is the most widely used illicit drug worldwide. A recent large systematic review has shown that cannabis may have therapeutic properties, prompting the campaign for its medical legalization [1]. Marijuana consumption among older US adults, in particular, has doubled since 2014 with edible formulations being more popular than inhaled cannabis [2]. Edible cannabis is associated with a high rate of emergency room visits due to intoxication, adverse reactions, and psychoactive effects. Cardiovascular adverse effects from oral formulations account for up to 20% of emergency room visits due to cannabis use [2]. Here, we present a case of accidental cannabis ingestion in an elderly male leading to a nearly fatal bradyarrhythmia.

## Case presentation

**Figure 1:**
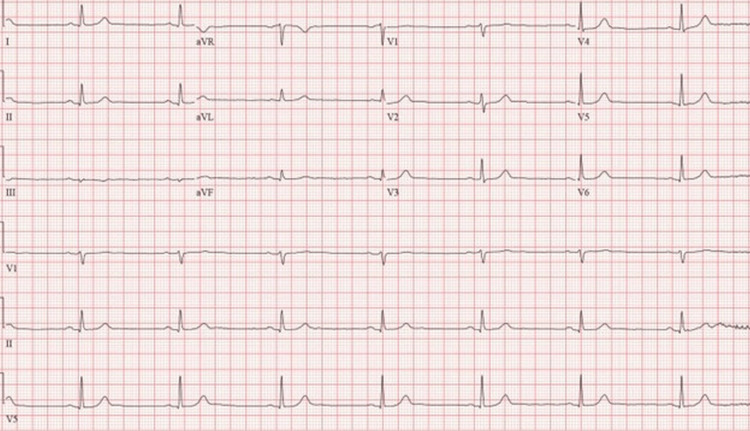
EKG on admission revealing sinus bradycardia with a heart rate of 45 beats per minute. EKG: electrocardiogram

A 74-year-old male presented for one day of dizziness and near syncope. The patient was spending time at his daughter’s house when he became progressively dizzy and altered throughout the day. His medical history was significant for hypertension and chronic obstructive pulmonary disease (COPD) due to a remote history of cigarette use. His only home medication was amlodipine 5 mg daily. The patient had no significant family medical history. In the emergency department, he was diaphoretic, dizzy, and slightly confused. A “stroke alert” was activated due to concern for a posterior circulation stroke. A computed tomography (CT) scan of the head and a CT angiography (CTA) scan of the head and neck were negative for acute intracranial abnormality. His initial troponins were within normal ranges. He was afebrile, with a blood pressure of 125/72 mmHg and oxygen saturation of 93% on room air. Vital signs were notable for sinus bradycardia with a heart rate of 30 beats per minute (bpm), which spontaneously resolved after one episode of nonbilious emesis to a heart rate of 45 bpm (Figure 1).

Hours after admission, the patient became unresponsive, and a hospital-wide alert was activated for concern of impending cardiac arrest. On bedside evaluation, the heart rate was 23 bpm and the blood pressure was 108/78 mmHg. The patient emergently received 0.5 milligrams (mg) of intravenous (IV) atropine one time and 1 liter (L) of IV normal saline, which resulted in an improvement in mental status to baseline. The patient’s laboratory work revealed no metabolic abnormalities that could contribute to presentation. His complete metabolic panel and complete blood counts were within normal limits. Telemetry evaluation revealed sinus bradycardia and a three-second sinus pause without evidence of atrioventricular (AV) blockade. The daughter then arrived at the bedside and stated that her father unknowingly consumed cannabis cookies at her house, which she purchased from a medical dispensary in Colorado. The recommended serving was one-eighth of a cookie; however, the patient accidentally consumed three entire cookies. The urine drug screen was only positive for tetrahydrocannabinol (THC).

**Figure 2:**
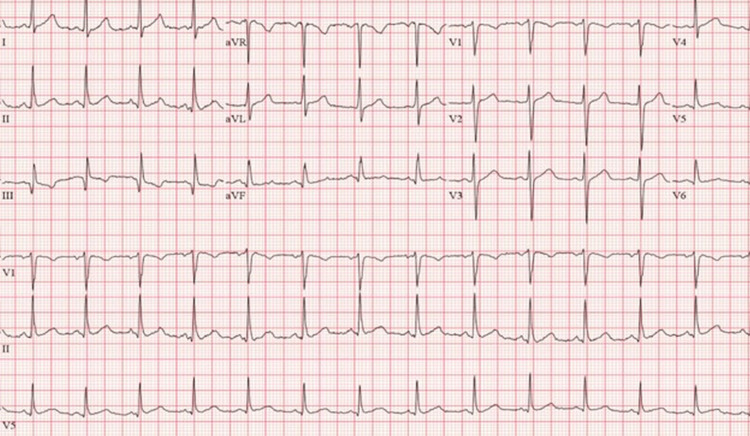
EKG on discharge showing normal sinus rhythm and a heart rate of 75 beats per minute. EKG: electrocardiogram

Cardiology was consulted to rule out the ischemic etiology of symptomatic bradycardia. Serial troponins were within normal limits, and the echocardiogram was normal with a left ventricular ejection fraction (EF) of 60%-64%. The nuclear medicine stress test was negative for ischemia. In total, the patient had three episodes of symptomatic bradycardia requiring bedside evaluation but only received atropine once. Three days after presentation, he was discharged with a 30-day event monitor that showed an average heart rate of 70 bpm with no significant tachycardia or bradyarrhythmias noted. His EKG was normal at discharge with a rate of 75 bpm (Figure 2), and the symptomatic bradycardia was therefore attributed to accidental cannabis toxicity.

## Discussion

With the recent campaign for the legalization of cannabis, edible formulations have become increasingly popular. Newer forms of cannabis can be up to 10 times more potent than older formulations, increasing the risk of cannabis-related adverse events [3]. These events are variable and include psychosis, nausea, vomiting, hypertension, and arrhythmias [1,4].

Cannabidiol (CBD) and tetrahydrocannabinol (THC) are the most commonly studied cannabis compounds. The cardiovascular effects of CBD appear to be dose-dependent. High concentrations of CBD, in particular, lead to bradycardia via adenosine receptor activation [5]. In contrast, low to moderate concentrations of cannabis activate the sympathetic nervous system and can lead to hypertension, tachycardia, and atrial fibrillation [4].

THC, on the other hand, acts on cannabinoid receptors and is responsible for the psychoactive effects of cannabis [5]. The onset of effects ranges from 30 minutes to greater than one hour after ingestion, depending on the drug formulation and composition of other food. For example, consuming a high-fat meal will increase the absorption of oral cannabinoids [6]. The variable absorption of oral THC and delayed onset of effects can lead to accidental overconsumption, especially in naive users. CBD and THC can remain in highly perfused tissues, including the heart, for days, which may be a contributing factor in the frequency and severity of cardiac adverse effects [5,6]. Although the adverse effects of cannabis are rare, oral cannabis can cause severe, potentially life-threatening bradycardia, especially in elderly populations [7].

## Conclusions

Cannabis has a public reputation as being a relatively benign drug, largely used for recreational purposes. With new claims regarding the therapeutic benefits of cannabis, prompting its medical legalization, more research should be conducted to assess the safety and dose-related effects. CBD and THC can remain in highly perfused tissues, including the heart, for days, which may be a contributing factor in the frequency and severity of cardiac adverse effects. This case highlights the impact of the recent legalization of marijuana on patients, especially the elderly, who may be prone to cardiovascular adverse effects. A comprehensive history and physical examination are vital in identifying accidental or purposeful ingestion of cannabis. Given the rapidly changing laws regarding cannabis use and increased access to new formulations, further investigation should be conducted into the cardiovascular adverse effects of oral cannabis.
